# Strengthening Global Health Research

**DOI:** 10.1080/16549716.2023.2290638

**Published:** 2023-12-22

**Authors:** Lars L. Gustafsson

**Affiliations:** Division of Clinical Pharmacology, Department of Laboratory Medicine, Karolinska Institutet at Karolinska University Hospital, Stockholm, Sweden

**Keywords:** Antibiotic resistance, capacity building, climate and health, development research, Global Health Research, sustainable development, transdisciplinary

## Abstract

Global Health is a young discipline with equity of health and services as its core value. The discipline has a tradition of close links between practice and research in line with the ‘Health for All’ declaration launched by the World Health Organization (WHO) in 1978. The multitude of existential health crises facing mankind require a research agenda in line with Global Health Research core values and methods, such as transdisciplinary collaboration, long time series of population-based observations and multifaceted interventions. Knowledge gaps cover climate effects on health and mechanisms for global spread and control of antibiotic resistance across species. Such health threats are preferably studied at Health and Demographic Surveillance Sites, a scientific infrastructure for Global Health Research in Africa and Asia, that gains to expand and monitor climate parameters and include sites in the northern hemisphere. Global Health Scientists together with science societies can ensure long-term funding of a global network of population-based health-climate sites. Global Health Scientists and scientific journals should jointly provide data and evidence on global health to governance bodies on regional, national and global levels, in particular to WHO and United Nations in charge of the programme with Sustainable Development Goals.

## Historical notes on global health

The global health core value is equity in health and services in line with the ‘Health for All’ model adopted by the World Health Organization (WHO) member states in Alma Ata in 1978 [1,2]. One of its fathers was the legendary WHO director Halfdan Mahler (1973–1988) [2]. A multifactorial disease concept grounded on a close link between practice and research replaced the one-dimensional biomedical disease model [2,3]. Health programmes and policy recommendations in member states and at WHO became solidly grounded on evidence and research findings [2]. Independent science advisory boards at WHO headquarter and at the six regional offices designed by Mahler and the Swedish Nobel Laureate Sune Bergstrom were formed to support programmes and leaders [4]. The global Tropical Disease Research and Human Reproduction programmes were founded to strengthen research capacity in low- and middle-income countries and became success stories [4]. The initiatives were possible through a global innovative funding model involving nations, foundations, the World Bank and WHO [4].

During the 1980s, Global Health Scientists and leaders argued that each and every country needs essential health research capacity to ensure that preventive and curative programmes accord to the needs of the country [4]. This philosophy was summarised as recommendations by the ‘Consortium on Health Research for Development’ 1990, an independent thinktank with global representation [4].

Global health issues are on the top of international political summits and integrated into the majority of the United Nations (UN) Sustainable Development Goals (SDGs) adopted by 193 member states in 2015 [5]. Still the world lacks trusted, transparent and effective global bodies for global health diplomacy [6,7,8]. It is urgent to strengthen the UN health diplomacy since no progress was registered up to Summer 2023 in achieving the agreed 17 SDG targets for the 17 SDGs [5,9,10].

How can the Global Health Research community contribute so health for all is tangible within a decade? Global Health Research should overcome its colonial heritage [11] and build trust on its core values and apply solid analytical transdisciplinary and multifaceted intervention approaches to generate comprehensive insights into health and clinical services.

## The anthropocene era and funding threats

A multitude of crises of unprecedented character (climate, Russian war in Ukraine, Palestine–Israel conflict, risks of pandemics, starvation and sky-rocketing living costs) are jeopardising sustainable and equitable health for all. Country after country are faced with nationalistic and populistic movements demanding cuts of foreign aid and development research and resisting funding of the green transition in their home countries or through multilateral organisations like UN [10,12,13].

United Kingdom’s government suddenly cut foreign aid by £4 billion (30%) in 2021, referring to a financial crisis caused by the COVID-19 pandemic [12]. A control programme of Neglected Tropical Diseases with an annual budget of £220 million to 23 African countries since 2008 was ceased immediately [12]. Recently, ‘Council on Health Research for Development’, which coordinates global health research hubs, closed its operations due to lack of funding [4]. During 2022, the Swedish government cut the development research budget for low- and middle-income countries from $90 to $45 million annually and stopped funding of development research projects at Swedish universities and coverage of Swedish expert assignments at international development organisations in 2023 [13]. The government simply announced that the needs of Ukraine was a priority [13]. A long Swedish era of generous development aid and support of UN and its expert organisations ended [13]!

## Examples of knowledge gaps

Global Health Research has a strong tradition of transdisciplinary approaches with long time series of data exploring the burden, the needs and possibilities for underprivileged populations [3,14]. A further strength is its tradition to collaborate across borders [15]. Global Health’s comprehensive research philosophy is needed to uncover knowledge gaps in a time with interconnected crises affecting public health.

The impact of climate change on health as well as an exponential rate of spread of antibiotic resistance are complex phenomena spanning molecular mechanisms to behavioural, social and economic perspectives. The present speed of fossil emissions is predicted to cause a 3°C increase above the preindustrial level of global temperature already in 2100 [16]. The annual cost of antibiotic resistance might reach 10 million deaths by 2050 [17]. Both these challenges require immediate actions, preventive long-term efforts, increasing awareness accompanied by changed behaviour and ground-breaking molecular and society-oriented research. Global Health researchers can contribute with expertise on design, management and evaluation of models for changed behaviour from individuals to societies.

The power of long time series to monitor climate and health parameters in a defined region was elegantly demonstrated in a study from a rural Burkina Faso Demographic Surveillance Site recently [18]. The researchers used population and climate data covering 15 years and verified that periods of extreme hot temperatures increased cause-specific deaths [18]. Today, there are a few other African sites that long-term monitor demographics, collect climate data (temperature, precipitation and wind), follow health parameters of the population with wearable devices (heart and ventilation rates, temperature, mobility and sleep) and verify health status with verbal autopsies [19]. The sites have started to explore geospatial events over time using satellite-collected sensed data, i.e. crop yields [19].

The health of the oceans is threatened by waste, fossil emissions and unsafe human practices, threatening daily life and businesses along the shores [20]. Examples of successful multifaceted interventions to handle the threats are reported such as the joint ‘2050 year strategy for the blue Pacific Continent’ involving 18 sovereign pacific states. This programme aims to preserve ocean health by alloying traditional conservational values with rules for high-tech surveillance of the health of the environment [20].

The increased prevalence of antibiotic resistance among bacterial strains in man, cattle and at poultries and fisheries is caused by inappropriate use of antibiotics [21,22,23]. The type and prevalence of antibiotic resistance varies, which is highly influenced by the total ‘antibiotic exposure’ in a region governed by behavioural, epidemiological and medical factors [22-23]. The research agenda should include factors governing how antibiotics are used, prevalence of molecular and clinical resistance and models for behavioural change involving all stakeholders. Research has to adhere to the fact that less than half of the use of antibiotics is in humans making the one-health approach spanning human, animal and fish perspectives mandatory [22]. It is to be noted that due to a comprehensive approach Global Health researchers have been pioneers in the field [22-23]. In fact, antibiotic research involves most disciplines and can be characterised as development research benefiting all types of societies.

## Scientific and methodological development

Scientific publications originate from few countries. So-called parasitism authorship (a publication without author from the country of study) is ethically unsound but frequently reported for studies pursued in countries like South of Sahara and published in North American journals [24]. It is positive that Global Health Action’s (GHA) succeeded to include Ethiopia, Indonesia and South Africa as top rated original corresponding countries for publications during GHA’s initial decade [25]. Global Health Journals should start joint efforts to train young scientists in scientific writing through online training opportunities combined with mentoring by senior scientists during the submission and review processes [26].

Global Health Research will increase its impact if more analytical and interventional cross-country studies on health, disease patterns and therapies are carried out. The global network of Health and Demographic Surveillance Sites (HDSS) started in the 1950s and is exemplary [27]. It now consists of more than 50 different sites across Africa and Asia [27]. HDSS units should expand their scope and include monitoring of climate parameters and establish units in the northern hemisphere. Such a Health-Climate Site Monitoring Network (HCSMN) can deliver health, climate and geospatial longitudinal data needed to address antibiotic resistance and climate effects on health. The Global Health Research community should argue for global funding of HCSMN as astronomers and physicists have succeeded to fund observatories and particle accelerators for decades. A tentative funder is the proposed global fund with 1 billion $ annual budget to finance global challenges as suggested by International Science Council (ISC) representing 250 national and global scientific societies and associations across disciplines [19,28-29]. This infrastructure can also serve as innovation hubs for health technologies.

I think that it is wise if Global Health Research increasingly includes laboratory research (molecular biological methods, sensitive nanotechniques for analysis of endogenous substances and drugs and microsampling non-invasive methods) with clinical, epidemiological and behavioural approaches [30]. Molecular methods are key components of clinical bacteriology, pharmacology and virology today and required for diagnosis, therapy and prevention of diseases. The neglected use of laboratory methods is illustrated by the fact that only 4 out of 1947 published articles in GHA (up to October 2023) were classified with the polymerase chain reaction (PCR) as MESH-term. PCR is an indispensable technique in the laboratory and is required for diagnosis of COVID-19 infection.

## Global impact and sustainable funding

Global Health Journals including Lancet Global Health and BMJ Global Health adhere to an open-access publishing policy that was pioneered by GHA at its launch in 2008 [14]. This has indeed favoured widespread availability of new knowledge and study results for scientists, policy staff, politicians and the public across the globe.

We need to reinstall the ‘Health for all’ model launched by WHO when scientists had advisory and strategic roles for executive health bodies and for managers. Presently, there seems to be a window of opportunity to ensure better connection between Global Health research and practice. UN Secretary General as well as the questioned SDG program have identified needs for longitudinal independent data on global health issues for optimal priorities and decisions [28,29,31]. This is a chance for Global Health Journals and leading Centers and Global Health Alliances to collaborate with ISC and play a key role for the provision of knowledge and evidence when the SDG programme is redirected. An apparent issue to address for such a global diplomacy network is to convince UN and WHO representatives about the need to fund a global research site infrastructure for the study and monitoring of the interaction between climate and health (HCSMN) [19,31,32].

## Paper context

Global Health has emerged as a concept in recent decades, inspired by the World Health Organization (WHO) movement ‘Health for all’ in the 1970s. Still Global Health has colonial roots to be addressed. Global Health Research can be strengthened by bridging Research and Development, a paradigm for multilateral aid during the 90s. Present existential global crises require multifaceted and cross-disciplinary solutions based on Global Health Research approaches.
